# Epoxidized Ionic Liquids as Processing Auxiliaries of Poly(Lactic Acid) Matrix: Influence on the Manufacture, Structural and Physical Properties

**DOI:** 10.3390/nano13091476

**Published:** 2023-04-26

**Authors:** Claudia Merlini, Vanessa Oliveira Castro, Gabriel Perli, Younes el Omari, Sébastien Livi

**Affiliations:** 1 Materials Engineering Special Coordination, Federal University of Santa Catarina (UFSC), Blumenau 89036-002, SC, Brazil; 2 Université de Lyon, CNRS, Université Claude Bernard Lyon 1, INSA Lyon, Université Jean Monnet, UMR 5223, Ingénierie des Matériaux Polymères, CEDEX F-69621 Villeurbanne, France; 3 Mechanical Engineering Department, Federal University of Santa Catarina, Florianopolis 88040-900, SC, Brazil

**Keywords:** ionic liquids, epoxy monomers, biodegradable polymers, processing auxiliaries

## Abstract

In this study, we set out to modify poly(lactic acid) (PLA) by incorporating epoxidized ionic liquids (ILs) that were specifically designed with imidazolium-NTf_2_ moieties. First, we synthesized di-, tri- and tetra-epoxidized ILs, which were incorporated into a PLA matrix at 3, 5, and 10 wt% through a melt extrusion process. We investigated the relationship between the structure and properties of the resulting materials in terms of thermal, mechanical, rheological, and surface properties. The results showed the potential of ILs to impact these properties. Notably, the tri- and tetra-epoxidized ILs enhanced the thermal stability of the PLA matrix as well as the crystallinity while reducing the glass transition temperature and melting point, which is promising for reactive extrusion processing. Overall, this research opens new routes for using reactive ILs to improve the processing and properties of PLA polymers.

## 1. Introduction

Poly(lactic acid) (PLA) is one of the most widely used biodegradable polymers and is presented as a good alternative to petroleum-derived plastics in several commodity applications [1]. PLA has the advantages of being eco-friendly, biodegradable, and biocompatible; it also presents high stiffness compared to other biodegradable polymers [2,3]. However, PLA has the major disadvantage of being brittle, which limits its applications in many fields. Some difficulties in PLA extrusion are related to its low toughness, high brittleness, low melt strength, low heat bending temperature, low thermal stability, narrow processing window, and non-conductivity. Moreover, PLA is well-known to be sensitive to higher temperatures during the melt process due to rapid degradation [1,2,4].

Many strategies have been developed in the literature to improve the ductility, and toughness of PLA by playing on the stereochemistry and crystallinity, adding different polymers, or by the use of plasticizers [5]. Plasticizers are frequently used, not only to improve the processability but also to increase the ductility of the PLA matrix. Moreover, a major challenge is related to the increase in molecular weight to improve the physical properties [6]. Furthermore, the higher the molecular weight, the broader the processing window to avoid PLA degradation [7,8]. The molecular weight can be improved through chain extenders that can connect the end groups (-OH) of PLA [9]. Among conventional plasticizers, phthalates are widely reported in the PLA matrix but their leaching and migration can generate potential health risks from chronic exposure [10,11].

Recent works demonstrated that ionic liquids (ILs) can be potential candidates for use as multifunctional chemicals that can increase molecular weight and flexibility [10,12,13,14,15]. In the studies reported by Park et al. [13,16], two phosphonium ILs combined with different counter anions (decanoate, tetrafluoroborate) employed at 5 wt% showed promising plasticizing behavior in PLA. Zhang et al. [10], reported the use of imidazolim IL 1-methyl-3-pentylimidazolium hexafluorophosphate [MPI][PF6] as a plasticizer for PLA. Other authors have reported PLA with improved mechanical performances by using imidazolium ILs [12].

In addition, ILs may also offer several advantages, such as low volatility and higher thermal stability, and lower solid–solid migration compared to molecular plasticizers [13]. Moreover, they are also emerging as a new class of ‘green plasticizers’ and they are an alternative to existing environmentally friendly materials [10,17,18,19].

Several anion-cation combinations of ILs result in a wide range of applications. Among these ILs, epoxidized Ils can be interesting agents because the epoxy group can react with both of the end groups, i.e., hydroxyl and carboxyl of the PLA matrix [15,20]. In this context, these ILs can enhance the flexibility and molecular weight and tune the mechanical properties of the PLA matrix. In this work, the effect of different types and amounts of ILs based on epoxy groups (di-, tri- and tetra-epoxidized) on PLA properties was investigated through reactive extrusion. The goal of this work is to investigate the application of reactive ILs as additives to adjust different properties of PLA.

## 2. Materials and Methods

**Figure 1:**
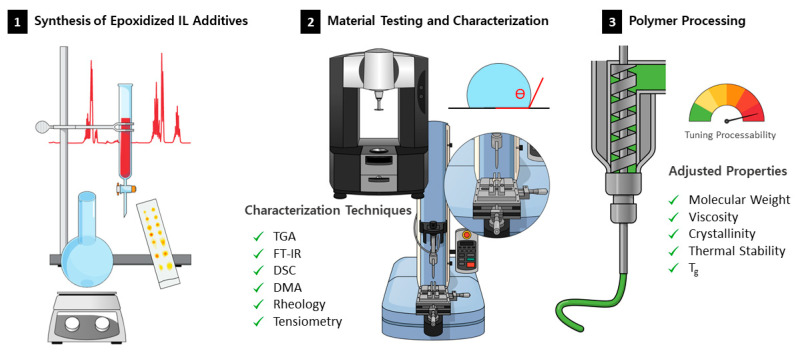
Diagram outlining the overall concept and procedure of this research. (**1**) Synthesis of the epoxidized ILs; (**2**) Examination of the polymer characteristics resulting from the incorporation of the epoxidized ILs; (**3**) Employment of the reactive extrusion to facilitate the tailored polymer properties.

In this study, three different epoxy monomers were designed bearing an imidazolium moiety and ester groups for the tri- and tetra-epoxidized. The objective was to explore the potential benefits of using these IL monomers as additives to modify the physical properties such as structural, thermal, mechanical, and surface properties. In addition, we sought to tailor the processing of the resulting PLA-derived polymers by adjusting their viscosity, crystallinity, and thermal stability. These aspects are crucial in the context of reactive extrusion, a common method for processing PLA (see Figure 1).

### 2.1. Materials

PLA (Ingeo™ Biopolymer 4043D) in pellet form, with a density of 1.24 g cm^−3^ and melt flow rate of 6 g‧10 min^−1^ (210 °C, 2.16 kg), was purchased from NatureWorks LLC (Savage, MN, USA). All reagents were purchased from Sigma Aldrich and were used without further purification. All the solvents including anhydrous solvents were purchased from Carlos Erba and used as received. The anhydrous-grade solvents were obtained in sealed flasks and used under a nitrogen atmosphere.

### 2.2. Synthesis of Di-, Tri- and Tetra-Epoxidized

**Scheme 1:**
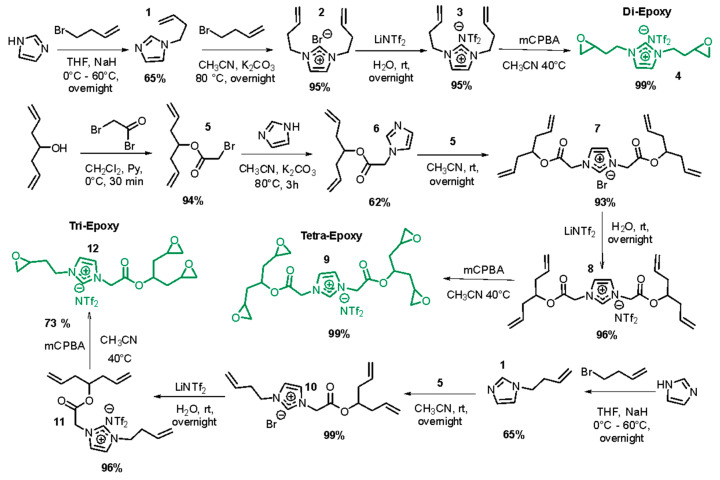
Synthetic routes employed for obtaining di-, tri-, and tetra-epoxies.

The imidazolium-based epoxy monomers were synthesized using the methodology outlined in recent publications from our group [17,21,22]. The three synthetic routes were optimized and afforded the synthesis of IL monomers in large quantities. Scheme 1 shows the synthetic pathway including the conditions and yields to obtain the epoxidized IL monomers. The imidazolium-based epoxy monomers were named di-epoxidized, tri-epoxidized, and tetra-epoxidized.

### 2.3. Extrusion Followed by Injection Molding of Samples

The PLA pellets were dried at 70 °C overnight before extrusion and injection molding. The PLA was mixed with IL and then incorporated into a twin-screw extruder, DSM Micro 15, with a screw speed of 70 rpm. During processing, the extrusion barrel temperature profile was fixed at 170 °C, 180 °C, and 180 °C for the feeding zone, compression zone, and die, respectively. After 3 min of mixing, at 70 rpm, the melted compound was fed into the barrel (at 200 °C) of the injection molding machine and then injected into the mold (mold temperature: 40 °C). The injection pressure was kept at 7 bar for 4 s, and 6 bar for 5 s. Injection-molded specimens were prepared using neat PLA, and PLA with 3, 5, and 10 wt% of different ILs: di-, tri- and tetra-epoxidized. The samples have been denoted as PLA/xy, where *x* represents the weight fraction of IL and *y* is the type (di-, tri-, or tetra-epoxidized).

### 2.4. Characterization

The number average molecular weight and average molecular weight of the PLA and PLA/ILs were determined by gel permeation chromatography (GPC) on a Light Scattering Instrument TREOS, Cell Type K5. Chloroform was used as eluent at a flow rate of 1.0 mL‧min^−1^.

Thermogravimetric Analysis (TGA) was carried out using a TGA550 thermogravimetric analyzer (TA Instruments, New Castle, DE, USA). The analyses were performed at 10 °C min^−1^ from 35 °C to 700 °C under a nitrogen flux of 60 mL‧min^−1^.

Fourier transform infrared spectroscopy (FT-IR) spectra were recorded on a Nicolet iS10 Thermo Scientific spectrometer in attenuated total reflectance mode (ATR). The spectra were collected for 32 scans with a spectral resolution of 4 cm^−1^ from 4000 to 500 cm^−1^, at room temperature (25 °C).

Differential Scanning Calorimetry (DSC) analyses were performed using a Q20 DSC (TA Instruments, New Castle, DE, USA). The samples were sealed in hermetic aluminum pans and heated from 25 to 200 °C (1st heating), followed by a cooling scan from 200 to 25 °C and then reheated to 200 °C (2nd heating), at a rate of 10 °C‧min^−1^, under nitrogen flow (60 mL‧min^−1^). The crystallinity content (*Xc*) was calculated based on Equation (1).

(1) $$Xc=\frac{\Delta H_{f}}{\Delta H_{f}^{*}\varphi }\cdot 100$$

where Δ*H_f_* is the sample enthalpy of fusion, ΔH_f_^*^ is the heat of fusion of perfectly crystalline PLA (93.7 J‧g^−1^) (4), and *φ* is the weight fraction of PLA.

The mechanical test was performed using an Instron 33R 4469 testing machine at room temperature (23 ± 1 °C). A cross-head speed of 10 mm/min was used for testing the dog-bone-shaped injection specimens (testing area: thickness 2 mm, width 4 mm, and length 30 mm). For each composition, five samples were tested.

The dynamic-mechanical properties of the PLA and PLA/IL were studied using a dynamic-mechanical analyzer (DMA 1 Star System from Mettler Toledo). The DMA measurements were carried out at a heating rate of 3 °C‧min^−1^, a frequency of 1 Hz, and a displacement of 2 µm, from 0 to 120 °C, in tensile mode on injected rectangular specimens with a thickness of 2 mm, a width of 4 mm, and a length of 30 mm. Nitrogen was used as the cooling agent.

The melt rheological properties of PLA and PLA/ILs were investigated under dynamical shear with an ARES-G2 rheometer (TA Instruments, New Castle, DE, USA). A parallel plate (Ø = 25 mm, gap = 1 mm) geometry was selected for the dynamic frequency sweeps under a controlled strain of 8%. This strain value was first verified to be in the linear viscoelastic region for all the evaluated samples. Frequency sweeps were performed from 100 to 0.1 rad/s at a temperature of 190 °C. To avoid the thermal degradation of the PLA, the heated chamber was purged constantly with nitrogen gas.

## 3. Results and Discussion

### 3.1. Increasing Molecular Weight (M_w_)

The average molecular weight (M_w_) and number average molecular weight (M_n_) of PLA and PLA/ILs are shown in Table 1. It is possible to note that with di-epoxide the PLA molecular weight (M_w_ and M_n_) tends to increase for fractions of 3 and 5 wt% of ILs, while for the highest amount of di-epoxide (10 wt%), the molecular weight of PLA decreases. This behavior can be related to the high amount of IL, which can result in an uneven distribution, leading to the formation of crosslinking or branching mostly of low molecular weight [6]. Conversely, by using tri- and tetra-epoxidized ILs, in all concentrations, the molecular weight slightly increases. This result indicates that tri- and tetra-epoxidized ILs can act as a chain extender, improving the molecular weight of PLA. This is a new method to increase the molecular weight of PLA, by using a non-toxic chain extender.

The molecular weight obtained for PLA (M_w_ 9.187 × 10^4^ g‧mol^−1^) is in agreement with the values reported in the literature [23]. However, according to [23], this molecular weight is too low and the PLA can display a brittle behavior. PLA should have at least ~10^5^ g‧mol^−1^ order of molecular weight to exhibit an acceptable level of mechanical properties. Thus, we can conclude that the use of 5 and 10 wt% of tri- and tetra-epoxidized ILs can be a successful way to slightly increase the PLA molecular weight.

### 3.2. Thermal Stability of PLA/IL Mixtures

**Figure 2:**
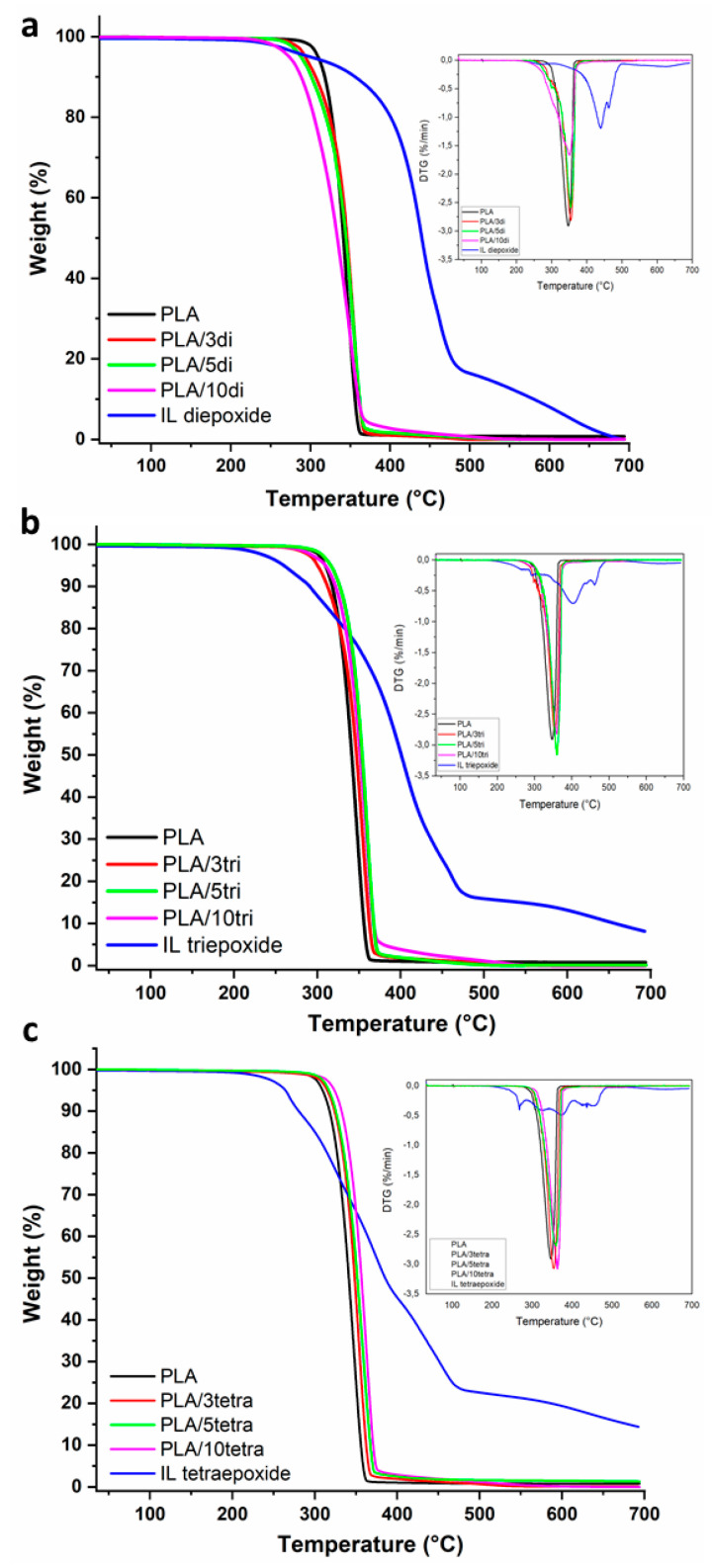
TGA and DTG curves of PLA with (**a**) di-, (**b**) tri-, and (**c**) tetra-epoxidized ILs.

The TGA and DTG thermograms of PLA and PLA with ILs are given in Figure 2. A detailed evaluation of the thermograms is presented in Table 2. The ILs presented thermal stability above 200 °C, indicating stability during the process performed in this work. A detailed description of the events can be found in Perli et al., 2022 [17]. In the TGA curves, it can be seen that PLA with and without ILs showed a one-step weight loss profile, with more than 98% of weight loss. The thermal stability of the polymer can be influenced by both the amount and type of IL present. When di-epoxide is used, the decomposition onset temperature (T_onset_) of PLA decreases, and this effect becomes more pronounced as the content of di-epoxide rises. The observed behavior may be attributed to the di-epoxidized IL having lower thermal stability, which causes it to undergo thermal decomposition at an earlier stage, and the less pronounced chemical interactions between the components. Meanwhile, the T_onset_ of PLA/tri and PLA/tetra was higher than neat PLA and PLA/di-epoxide (+0–25 °C). This behavior can be associated with the presence of the ester group in the tri- and tetra-epoxidized ILs that facilitates chemical interactions with PLA, improving thermal stability. In all samples containing ILs, the maximum decomposition temperature (T_max_) is observed to shift towards higher temperatures when compared to neat PLA.

### 3.3. Study of Molecular Interactions between ILs and PLA Matrices by FT-IR

**Figure 3:**
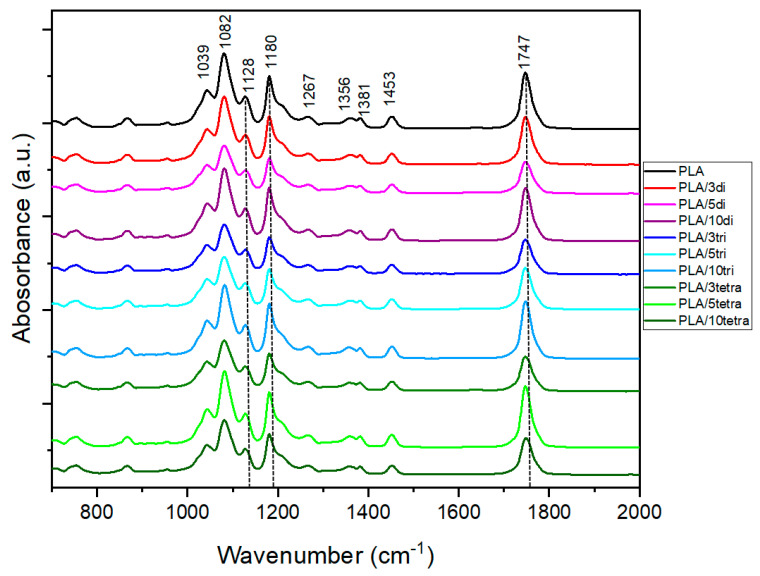
ATR-FT-IR spectra of PLA with and without ILs.

Figure 3 shows the ATR-FT-IR spectra of the PLA with and without epoxidized ILs, with their characteristic spectral bands. PLA shows characteristic bands of alkyl, carboxylic, ester, and ether groups, as described in detail in Table 3. The spectra of PLA with different types and amounts of IL show nearly the same absorption peaks as neat PLA. It is possible to observe that the bands of the C=O (1747 cm^−1^) and C-O groups (1128 and 1180 cm^−1^) of PLA with tri- and tetra-epoxidized ILs are displaced to lower wavenumbers. The observed behavior supports the existence of intermolecular interactions between the tri- and tetra-epoxidized ILs and PLA polymer, which are likely to involve hydrogen bonding. Specifically, it is hypothesized that a hydrogen bond is formed between the acid hydrogen from imidazolium and the ester group of PLA, which acts as a hydrogen bond acceptor. Furthermore, the epoxide groups can react with carboxyl or hydroxyl chain end groups of PLA [20]. Thus, the presence of three and four epoxide groups in the tri- and tetra-epoxidized, respectively increase the functionality of the IL, when compared to di-epoxide, and increase the likelihood of observing additional chemical interactions. Moreover, the presence of ester groups in the tri- and tetra-epoxide ILs can also lead to interactions with the hydroxyl groups of PLA.

### 3.4. Preliminary Investigation of Molecular Transitions and Crystallinity

The DSC thermograms of neat PLA and PLA/ILs during the second heating cycle are depicted in Supplementary Materials Figure S1. Within the experimental temperature range, all samples exhibit endothermic transitions such as glass transition temperature (T_g_) and melt temperature (T_m_), as well as an exothermic transition–cold crystallization temperature (T_cc_), as specified in Table 4.

In the first heating, the T_g_ for PLA was observed at 60.7 °C, and this value was reduced in the samples with ILs, indicating an increment in the polymer molecular mobility. A more significant reduction in T_g_ is observed with higher amounts of ILs. The second transition (exothermic) was observed for all compositions at about 112 °C, related to the cold crystallization of PLA. This behavior indicates that rapid cooling during the injection process prevented maximum polymer crystallization. Under these process conditions, a large number of crystal nuclei are formed, and thus, during further heating of the sample, the crystalline nuclei grow, giving rise to a rapid recrystallization, observed at temperatures lower than the melting point of the polymer.

The endothermic peak observed at around 150 °C is associated with the melting of the PLA crystallites. The addition of the three IL co-monomers causes a reduction in the melting temperature of PLA. Moreover, for almost all compositions (except PLA/3tetra) there is also a shoulder peak at around 152 °C, indicating that ILs can induce the formation of different crystalline phases. According to the literature, double melting transitions are frequently observed in PLA due to its polymorphic structure—the *α*-form melts at a higher temperature than the *β*-form [24,25]. At the same time, some authors have reported that the double peaks in PLA could be also related to crystal reorganization upon melting of imperfect crystals, formed in cold crystallization [25,26]. These crystals would have a high tendency to reorganize into more ordered structures and melt at higher temperatures. The crystallinity degrees of the samples with ILs are higher than that of neat PLA and increase with increasing amounts of IL, with higher values for PLA/IL with 10 wt% of ILs di-, tri-, and tetra-epoxidized. The absence of a crystallization peak on cooling confirms that the PLA crystallization does not occur under the test cooling conditions, as reported by other authors [25,26].

In the second heating, any previous thermal history is not present and it is possible to analyze separately the effect of the ILs on the thermal properties of the polymer. It is possible to note that T_g_ is reduced in all compositions containing ILs. This result indicates that the ILs can provide greater flexibility in the polymer chain, and thus, less energy is needed for the movement of the amorphous phase chains. Concerning T_m_, the behavior of samples of PLA/ILs was similar to those observed in the first heating, where PLA/IL displays lower T_m_. This behavior can be related to the presence of the IL, which reduces the intermolecular interactions between the chains in the crystalline phase, requiring less energy for melting to take place. With regard to crystallinity, the addition of ILs led to an increase in the crystallinity of PLA. Adjusting the degradability of PLA is a significant challenge and reducing the amorphous phase can have a significant effect on this degradation process. The observed behavior implies that ILs can improve the organization of PLA chains and potentially adjust the formation of the polymer’s crystalline phase. In general, incorporating IL co-monomers into PLA resulted in a decrease in the material’s melting temperatures and an increase in its degree of crystallinity. This could potentially lead to significant energy savings on an industrial scale and improved mechanical and degradation properties.

### 3.5. Modifying Mechanical Properties

**Figure 4:**
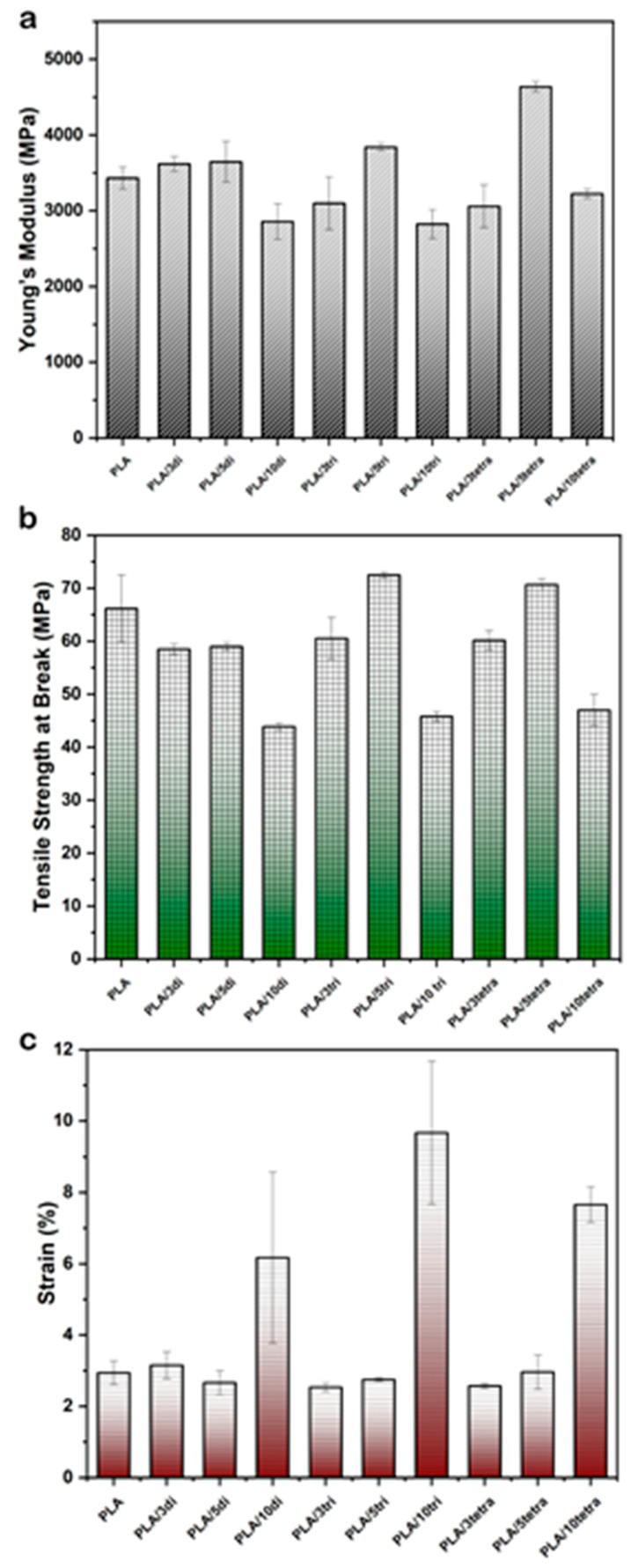
(**a**) Young’s modulus, (**b**) tensile strength, and (**c**) strain of PLA and PLA/IL samples.

Figure 4 displays the outcomes of the tensile tests performed on both PLA and PLA/ILs. Interestingly, the utilization of tri- and tetra-epoxidized ILs at a concentration of 5 wt% remarkably increased the Young Moduli and tensile strength at break of the resulting PLA/IL blends. This effect can be attributed to the rise in molecular weight, which results in increased chain entanglement, thereby leading to an enhancement in the Young’s modulus and tensile strength at break at room temperature. Basically, the tri- and tetra-epoxidized ILs act as chain extenders, raising the molecular weight and requiring more energy to break or loosen the entangled chains. Not only is this rationale supported by the observed increase in molecular weight, but it is also corroborated by the slight rise in both thermal stability and dynamic moduli.

Figure 4c highlights another interesting trend, wherein a significant alteration in the mechanical properties of PLA is observed upon the addition of 10 wt% of di-, tri-, and tetra-epoxidized ILs. These specimens exhibit an increase in ductility, as evidenced by the rise in PLA strain. These findings are of great interest, as they demonstrate that the flexibility of PLA can be adjusted by varying the amount and nature of IL additives, which opens up the possibility for diverse applications.

### 3.6. The Effect on Thermomechanical Properties

**Figure 5:**
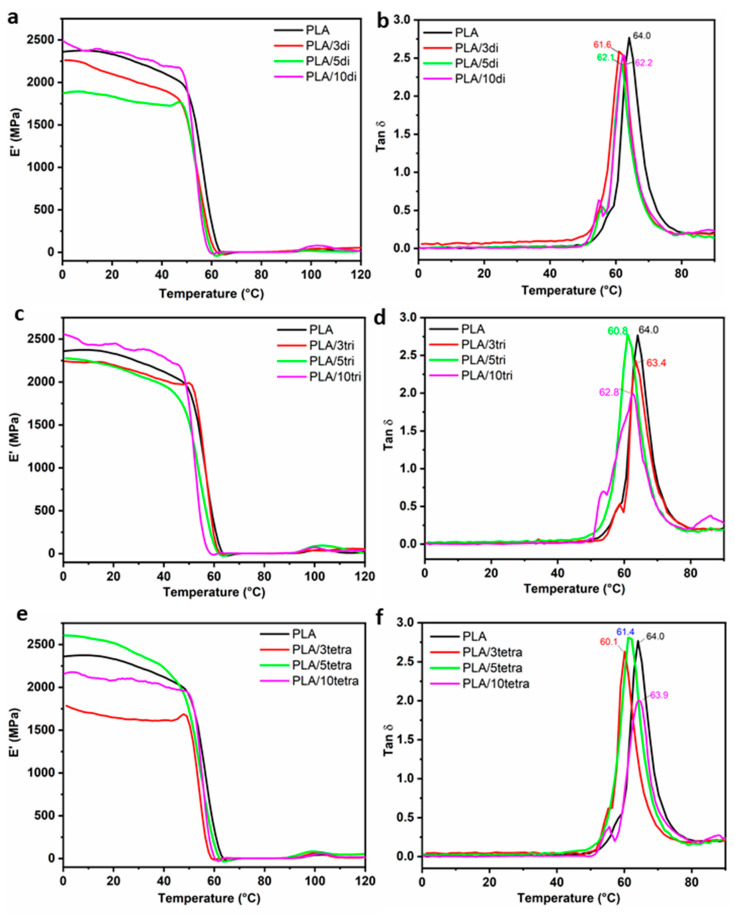
DMTA traces of the storage modulus and loss factor of PLA and PLA/ILs for (**a**,**b**) PLA/di-, (**c**,**d**) PLA/tri-, and (**e**,**f**) PLA/tetra-epoxidized ILs.

The storage modulus (E′) and loss tangent (tan δ) as a function of temperature of PLA and PLA/ILs are reported in Figure 5. Interestingly, small amounts of di-, tri-, and tetra-epoxidized tend to slightly decrease the elastic moduli at the glassy region preserving the same magnitude at the rubbery state. More importantly, increasing the percentage up to 10% for di- and tri-epoxides enhanced the elastic moduli at lower temperatures and for tetra-epoxide, the highest values were observed for the percentage of 5%.

For PLA/ILs with di-, tri-, and tetra-epoxidized ILs, the E′ values are lower than those found for the unmodified polymers over the whole temperature range. However, in the samples with higher amounts of ILs, there is a small increase in the E′. The transition temperature in the tan δ thermogram at around 64 °C corresponds to the glass transition (T_g_) of PLA. For all compositions, the T_g_ values are shifted to lower temperatures when different amounts and types of ILs were incorporated into the polymer. This behavior agrees with the results obtained in the DSC and mechanical tests, indicating that ILs can improve the flexibility of the polymer chains.

### 3.7. Adjusting the Viscosity in the Molten State

**Figure 6:**
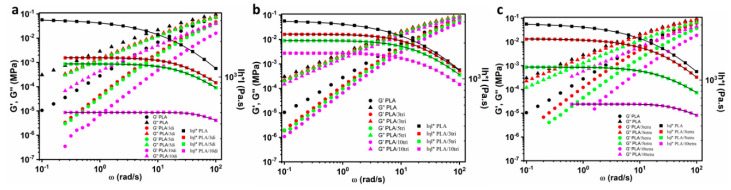
Dynamic modulus and Complex viscosities of neat and modified PLAs at 190 °C under 8% dynamic strain: (**a**) di-, (**b**) tri-, and (**c**) tetra-epoxidized ILs.

The elastic modulus G′, loss modulus G″, dynamic viscosity (|η*|), and the viscosity fitting of the Carreau Yasuda model (black curves) [27] for the PLA/ILs samples are illustrated in Figure 6. The PLA and PLA/ILs melt behaved as Newtonian fluids at low frequency and non-Newtonian fluids at high frequency. The dynamic viscosity decreased with the increase in shear rate, showing shear-thinning rheological behavior. As shown in Table 5, the higher the content of IL, the more Newtonian the material (the flow index n increases), while the zero-shear viscosity decreases. According to the literature, PLA can be modified depending on the requested property. Reignier and co-authors used Joncryl as a chain extender to improve the elasticity to improve the foamability of the PLA [28]. In the present work, the ILs play a plasticizer role in the molten state, which agrees with the DSC results. A reduction in T_g_ was observed with the increasing amount of ILs. This property might be attractive during processing when a medium viscosity is required, especially when the material has similar mechanical properties at room temperature.

### 3.8. Hypothetical Mechanisms

**Figure 7:**
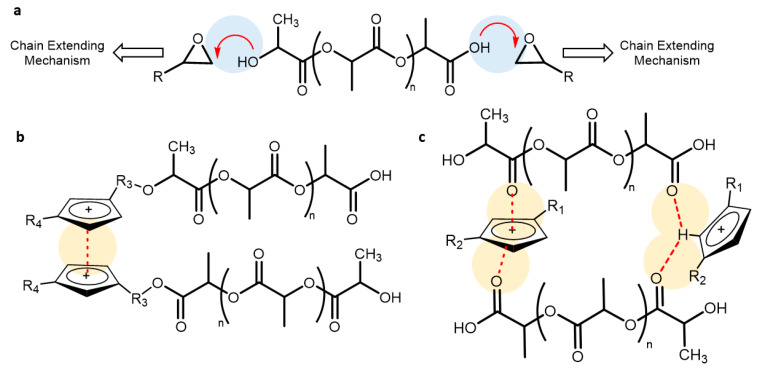
Proposed Mechanisms for Modifying PLA Properties. (**a**) the reaction of epoxy groups with hydroxyl and carboxyl terminal groups; (**b**) the π-π interaction between imidazolium moieties; and (**c**) potential ion-dipole and hydrogen bond interactions involving PLA carbonyl groups.

The goal of this work is to investigate and shed some light on the application of reactive ILs as additives to tailor different properties of PLA polymers. Upon examining the results, we observed that the ILs, mainly tri- and tetra-epoxidized, acted depending on the proportions as chain extenders, plasticizers, and agents that coordinate the formation of crystals. The epoxy groups tend to react with the hydroxyl and carboxyl termini through a nucleophilic attack extending the length of the PLA chains. This result is supported by the GPC outcomes, thermal stability, and the gain in the mechanical properties when tri- and tetra-epoxidized ILs were used in the proportion of 5 wt%. Consequently, following the reaction between the PLA terminal groups and ILs, the imidazolium group becomes a pendant group of the PLA chain and is capable of π-π stacking, as depicted in Figure 7.

It is widely recognized that intermolecular interactions among the constituents of a polymer can improve and customize its mechanical properties. Using FT-IR, we noted a decrease in the wavelength of the carbonyl vibrational modes, particularly for stretching mode, suggesting that the carbonyl groups in PLA engage in hydrogen bond interactions or ion-dipole interactions, as shown in Figure 7. Furthermore, these intermolecular interactions might facilitate the creation of more crystals, albeit imperfect ones, that could account for the rise in the degree of crystallinity but the decrease in the melting point.

The plasticizing effect is verified by the decrease in both the glass transition and alpha transition temperatures, indicating that not all of the ILs have reacted with the PLA terminal groups, depending on the IL content, and some of the molecules remain unattached in the PLA matrices. The reduction in both the glass transition and alpha transition temperatures confirms the occurrence of a plasticizing effect, which is dependent on the quantity of ILs added and the extent of their reaction with the PLA terminal groups, with some IL molecules remaining unattached in the PLA matrices. Undoubtedly, further investigation is necessary to substantiate these hypotheses. Nonetheless, in this study, our objective was to establish a basis for utilizing reactive epoxidized ILs for adjusting various polymer properties and comprehending the structure-property correlation.

## 4. Conclusions

In this research, we developed poly(lactic acid) (PLA) modified with di-, tri-, and tetra-epoxidized ILs using an extrusion mixture followed by injection molding. The addition of ionic liquids had a plasticizing effect on the PLA in the molten state, reducing the zero-shear viscosity and increasing the molecular mobility of the polymer, particularly at higher levels of ILs. We observed a chemical interaction between the tri- and tetra-epoxidized ILs, which resulted in an increase in the thermal stability of PLA. Interestingly, higher amounts of ILs led to a slight increase in the molecular weight of the polymer, which could broaden the processing window and prevent degradation. Our results suggest that tri- and tetra-epoxidized ILs may be more desirable as multifunctional additives for PLA than di-epoxide, offering a better balance between processing and properties. In future studies, we plan to perform soil degradation tests on PLA/ILs to further understand the effect of ILs. Overall, these findings highlight the potential of epoxidized ILs as a promising approach for modifying PLA properties and improving the processing of this important biopolymer.

## Figures and Tables

**Table 1:** Molecular weight of PLA and PLA/ILs.

Sample	M_w_ (g‧mol^−1^)	M_n_ (g‧mol^−1^)	Polydispersity (M_w_/M_n_)
PLA	9.187 × 10^4^	6.223 × 10^4^	1.476
PLA/3di	9.554 × 10^4^	7.020 × 10^4^	1.361
PLA/5di	1.004 × 10^5^	7.517 × 10^4^	1.336
PLA/10di	5.364 × 10^4^	4.928 × 10^4^	1.089
PLA/3tri	9.807 × 10^4^	7.479 × 10^4^	1.311
PLA/5tri	1.022 × 10^5^	7.861 × 10^4^	1.300
PLA/10tri	1.051 × 10^5^	8.753 × 10^4^	1.200
PLA/3tetra	9.657 × 10^4^	7.107 × 10^4^	1.359
PLA/5tetra	1.055 × 10^5^	8.706 × 10^4^	1.212
PLA/10tetra	1.026 × 10^5^	8.358 × 10^4^	1.228

**Table 2:** Thermal degradation temperatures of PLA and PLA/ILs.

Samples	T_onset_ (°C)	T_peak_ (°C)
PLA	319	339
PLA/3di	319	344
PLA/5di	309	343
PLA/10di	280	349
PLA/3tri	320	344
PLA/5tri	323	358
PLA/10tri	317	359
PLA/3tetra	328	345
PLA/5tetra	319	358
PLA/10tetra	327	362

**Table 3:** Wavenumbers and their corresponding vibrational assignments for PLA FT-IR spectra (1,2).

Wavenumbers (cm^−1^)	Assignments	Type of Vibration *
1039	C–CH_3_	st
1082, 1128, 1180	C–O	st (as)
1267	C=O	δ
1356, 1381	CH	δ (s and as)
1453	CH_3_	δ (as)
1747	C=O	st
2947, 2994	CH_3_	st (as and s)

* st, stretching; δ, bending; s, symmetrical; as, asymmetrical.

**Table 4:** Thermal properties of PLA and PLA/ILs in the 1st and 2nd heating cycles.

Samples	T_g_		T_cc_		T_m_		ΔH_m_		X_c_ (%)	
	1st	2nd	1st	2nd	1st	2nd	1st	2nd	1st	2nd
PLA	60	60	112	116	150	150	25	24	27	26
PLA/3di	59	57	113	114	148	148	25	25	27	28
PLA/5di	58	57	112	115	148	148	25	24	28	27
PLA/10di	57	54	109	112	147	147	30	31	35	37
PLA/3tri	59	56	112	113	149	147	26	25	28	28
PLA/5tri	58	55	111	111	148	146	29	30	33	34
PLA/10tri	55	57	112	115	148	147	31	30	37	35
PLA/3tetra	58	57	114	117	150	149	23	24	26	27
PLA/5tetra	55	57	106	114	149	141	28	29	32	33
PLA/10tetra	54	56	112	114	148	148	29	29	35	35

The transition enthalpies and temperatures are expressed in J‧g^−1^ and °C, respectively; 1st and 2nd mean first and second cycle, respectively.

**Table 5:** Zero-shear viscosities and flow behavior indexes of neat and modified PLAs at 190 °C.

Material	Zero-Shear Viscosity (Pa‧s)	Flow Behaviour Index
PLA	3093	0.64
PLA/3di	1458	0.70
PLA/5di	1293	0.73
PLA/10di	501	0.82
PLA/3tri	2372	0.59
PLA/5tri	2097	0.62
PLA/10tri	1665	0.61
PLA/3tetra	2277	0.65
PLA/5tetra	1297	0.84
PLA/10tetra	619	0.80

## Supplementary Materials

The following supporting information can be downloaded at: https://www.mdpi.com/article/10.3390/nano13091476/s1, Figure S1: DSC curves with 1st heating and 2nd heating of PLA with different ILs: (a) PLA/di, (b) PLA/tri and (c) PLA/tetra.
