# Supplementary material for: Epoxidized Ionic Liquids as Processing Auxiliaries of Poly(Lactic Acid) Matrix: Influence on the Manufacture, Structural and Physical Properties
Source: Nanomaterials (Basel). 2023 Apr 26;13(9):1476. doi: 10.3390/nano13091476 (PMC10180299; doi:10.3390/nano13091476)
Supplement: Supplementary file 1 [file nanomaterials-13-01476-s001.zip › nanomaterials-2336075-supplementary.pdf]

nanomaterials

Supplementary Materials

Epoxidized Ionic Liquids as Processing Auxiliaries of Poly(Lactic Acid) Matrix: Influence on the Manufacture, Structural and Physical Properties

Claudia Merlini [1,2], Vanessa Oliveira Castro [2,3], Gabriel Perli [2], Younes El Omari [2] and Sébastien Livi [2,*]

1 Materials Engineering Special Coordination, Federal University of Santa Catarina (UFSC), Blumenau 89036-002, SC, Brazil
2 Université de Lyon, CNRS, Université Claude Bernard Lyon 1, INSA Lyon, Université Jean Monnet, UMR 5223, Ingénierie des Matériaux Polymères, CEDEX F-69621 Villeurbanne, France
3 Mechanical Engineering Department, Federal University of Santa Catarina, Florianopolis 88040-900, SC, Brazil
* Correspondence: sebastien.livi@insa-lyon.fr

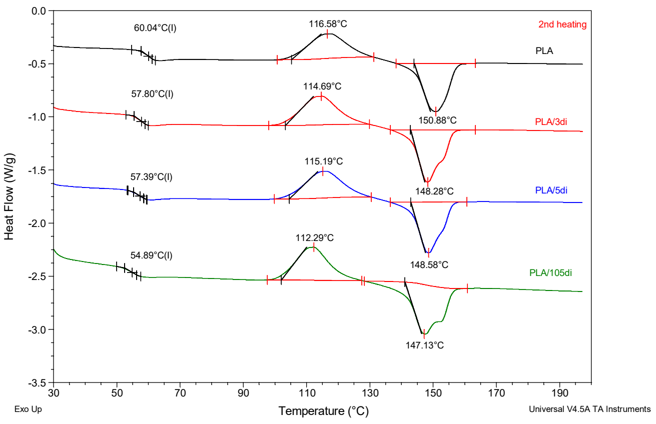

(**a**)

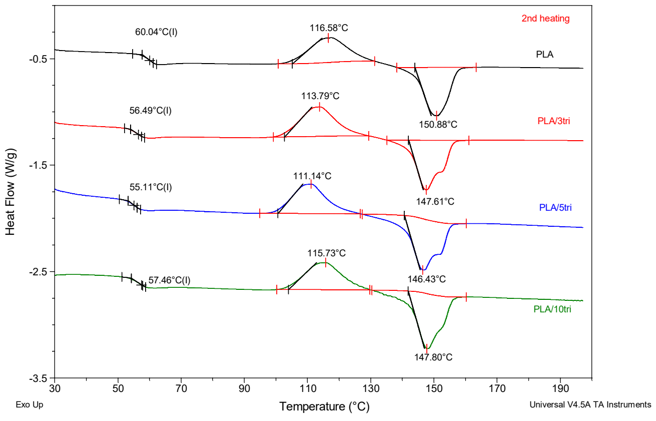

(**b**)

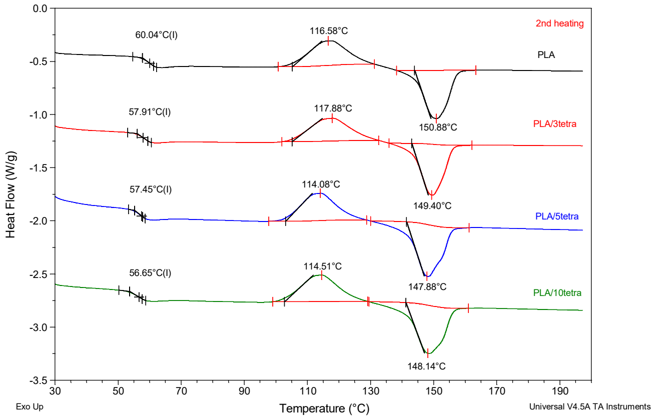

(**c**)

Figure S1. DSC curves with 1st heating and 2nd heating of PLA with different ILs: (**a**) PLA/di, (**b**) PLA/tri and (**c**) PLA/tetra.
